# *Yersinia pestis* Evolution on a Small Timescale: Comparison of Whole Genome Sequences from North America

**DOI:** 10.1371/journal.pone.0000770

**Published:** 2007-08-22

**Authors:** Raymond K. Auerbach, Apichai Tuanyok, William S. Probert, Leo Kenefic, Amy J. Vogler, David C. Bruce, Christine Munk, Thomas S. Brettin, Mark Eppinger, Jacques Ravel, David M. Wagner, Paul Keim

**Affiliations:** 1 Department of Biological Sciences, Northern Arizona University, Flagstaff, Arizona, United States of America; 2 Bioscience Division, Los Alamos National Laboratory, Los Alamos, New Mexico, United States of America; 3 Microbial Diseases Laboratory, California Department of Public Health, Richmond, California, United States of America; 4 Pathogen Genomics Division, Translational Genomics Research Institute, Phoenix, Arizona, United States of America; 5 J. Craig Venter Institute, The Institute for Genomic Research, Rockville, Maryland, United States of America; University of Maryland, United States of America

## Abstract

**Background:**

*Yersinia pestis*, the etiologic agent of plague, was responsible for several devastating epidemics throughout history and is currently of global importance to current public heath and biodefense efforts. *Y. pestis* is widespread in the Western United States. Because *Y. pestis* was first introduced to this region just over 100 years ago, there has been little time for genetic diversity to accumulate. Recent studies based upon single nucleotide polymorphisms have begun to quantify the genetic diversity of *Y. pestis* in North America.

**Methodology/Principal Findings:**

To examine the evolution of *Y. pestis* in North America, a gapped genome sequence of CA88-4125 was generated. Sequence comparison with another North American *Y. pestis* strain, CO92, identified seven regions of difference (six inversions, one rearrangement), differing IS element copy numbers, and several SNPs.

**Conclusions/Significance:**

The relatively large number of inverted/rearranged segments suggests that North American *Y. pestis* strains may be undergoing inversion fixation at high rates over a short time span, contributing to higher-than-expected diversity in this region. These findings will hopefully encourage the scientific community to sequence additional *Y. pestis* strains from North America and abroad, leading to a greater understanding of the evolutionary history of this pathogen.

## Introduction

*Yersinia pestis* is a Gram-negative bacterium and the causative agent of plague, a disease with global importance to public health and to biodefense efforts. *Y. pestis* is thought to have been responsible for three pandemics throughout history. The first pandemic, or Justinian's plague, occurred in Europe during the 6th century. The second pandemic lasted from the 14th to the 17th centuries and includes the Black Death that reduced Europe's population by 30–40% [1]. We are currently living within the third pandemic, or modern plague, which began in the 19th century when the disease emerged from Eastern China and was spread throughout the world via steamships. All continents except Australia and Antarctica currently possess active plague foci [1]. *Y. pestis* is an obligate pathogen that is found exclusively in arthropod vectors or mammalian hosts. The bacterium was developed as a biological weapon by the United States, the former Soviet Union, and Japan during the 20th century. It is currently classified as a Category A Select Agent by the US Centers for Disease Control and Prevention [2]. Although most *Y. pestis* infections are easily treated with antibiotics, an antibiotic-resistant strain has been discovered recently in Madagascar and fixation of such strains could pose a significant public health risk [3].

*Y. pestis* is a recently emerged clone of *Yersinia pseudotuberculosis*, evolving within the last 9,000–40,000 years [4], [5]. *Y. pestis* nomenclature was originally based upon differing biochemical characteristics, dividing strains into four biovars: Orientalis, Medievalis, Antiqua, and Microtus [6]. As new analysis methods emerged, these biovar groupings were found to inadequately reflect molecular relatedness among *Y. pestis* strains. As a result, a new nomenclature based on molecular relatedness was developed that incorporated the traditional biovar designations. The *Y. pestis* phylogeny currently has three major branches. Branch 0 contains almost all pestoides isolates and the Microtus isolate 91001 (groups 0.PE1, 0.PE2, 0.PE3, 0.PE4). Branch 1 contains all of Orientalis (1.ORI) and African Antiqua (1.ANT). Branch 2 contains all of Medievalis (2.MED) and Asian Antiqua (2.ANT) [5].

1.ORI spread throughout the world during the Third Pandemic and is the only *Y. pestis* type found in North America, having been introduced to this region within the last 125 years. A single synapomorphic SNP has been found for North American *Y. pestis*, supporting the hypothesis that North American plague is the result of a single introduction [Vogler et al, unpublished data]. In North America, plague was first documented in non-native rat populations in the 1890s and 1900s in Los Angeles, San Francisco, and Galveston [7]. Plague then largely disappeared from these cities due to improved hygiene and efforts to control the non-native rat population; however it reemerged in native rat populations and was responsible for an epidemic near San Francisco in 1908 [7]. After this initial introduction into native fauna, plague spread rapidly eastward through rodent populations. By 1950, *Y. pestis* reached its current North American distribution which includes the 17 westernmost states and a boundary of approximately 100°W longitude [8].

This note will address our early findings regarding *Y. pestis* CA88-4125 (GenBank accession: ABCD00000000), a strain isolated from a human case at Fort Hunter Liggett in Monterey County, California, in 1988. The California Department of Health ID is 88A-4125. Annotation of CA88 is currently in progress at the Enteropathogen Resource Integration Center (ERIC) and the contig sequences will soon be released into GenBank. Because plague was first introduced into North America through San Francisco, comparing CA88 to other North American Y. pestis strains may offer a glimpse into how plague has evolved as it spread eastward in this region. The CO92 genome sequence was closed and released in 2001 [9]. CO92 is a clinical isolate from Chafee County, Colorado, which was isolated in 1992. FV-1, a strain isolated from a natural outbreak near Flagstaff, Arizona, in 2001 [10], is also currently available in GenBank as 400 contig sequences [11]. CO92, FV-1, and CA88 are all members of biovar Orientalis and the 1.ORI branch. This note examines large-scale genomic differences between CA88 and CO92, two strains that are potentially very divergent among North American *Y. pestis*. Analyses were conducted *in silico* and indicate that North American *Y. pestis* have undergone rapid evolution in a very short time period.

## Results and Discussion

### CA88 contigs

The current CA88 sequence consists of ten contigs, three of which are from the three *Y. pestis* plasmids. The seven contigs from the chromosome total 4,650,262 bp, range from 43,407 to 1,676,077 bp in length, and have an average contig size of 664,323 bp.

### Chromosomal inversions

**Figure 1:**
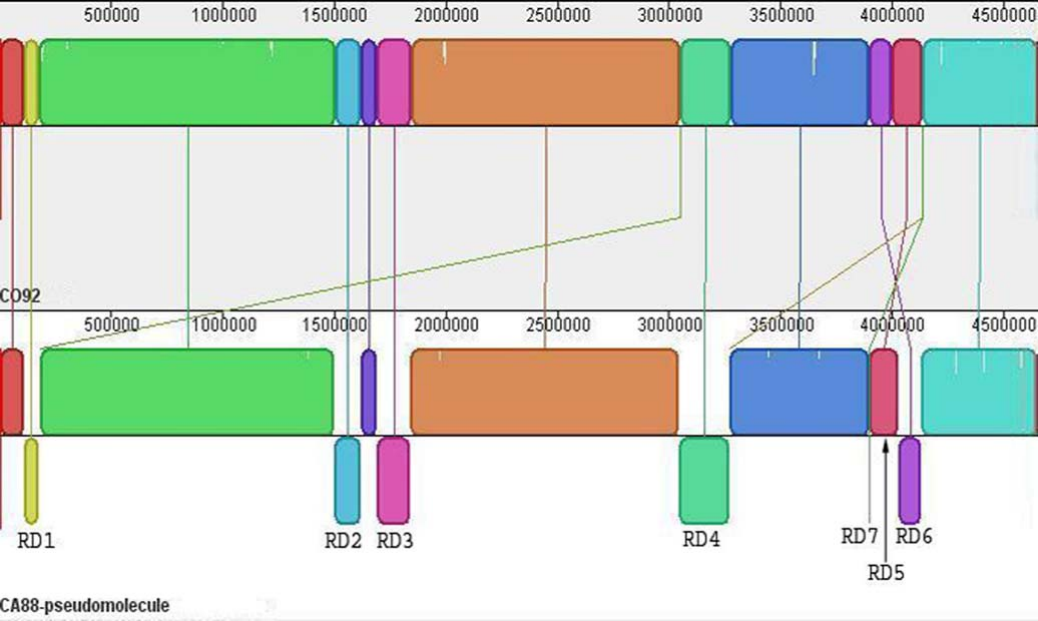
A whole-genome comparison between *Y. pestis* strains CO92 (top) and CA88 (bottom) using MAUVE. MAUVE found 15 LCBs shared between CO92 and CA88, six of which are inverted and one of which is rearranged. The seven large regions of difference are indicated. Some LCBs are too small to display on this figure and are shown by long diagonal lines connecting the genomes.

Fifteen shared local collinear blocks (LCBs) were determined, six of which were inverted and one of which was rearranged between CO92 and CA88 (Figure 1). These seven large regions of difference (RDs) are annotated in Table 1. Each of these regions is flanked by transposases on each side of the LCB, indicating a probable mechanism by which these rearrangements occurred [12]. None of these regions represent a contig in its entirety, reducing the chance that these rearrangements are the result of incorrect pseudomolecule assembly.

**Table 1:** Characteristics of the six inversions and one large rearranged LCB found via MAUVE.

Region of Difference	Type	CO92 Positions	LCB Length (bp)	Mechanism
1	Inversion	107,537–178,375	70,838	Transposase
2	Inversion	1,504,695–1,620,416	115,721	Transposase
3	Inversion	1,693,931–1,843,741	149,810	Transposase
4	Inversion	3,050,889–3,277,041	226,152	Transposase
5	Inversion and Rearrangement with RD6	3,900,833–4,000,718	99,885	Transposase
6	Rearrangement with RD5	4,000,718–4,136,714	135,996	Transposase
7	Inversion	4,136,720–4,137,899	1,179	Transposase

In addition to flanking transposases, several insertion (IS) elements of varying types and families were found within each LCB. These insertion elements could promote further rearrangement within these shared LCBs as time progresses and as the strains continue to diverge. This finding is consistent with a previous comparison between *Y. pestis* CO92 and *Y. pestis* KIM in which a large amount of genome rearrangement was observed. *Y. pestis* KIM is a member of biovar Medievalis and the 2.MED branch [5]. The rearrangements between CO92 and KIM were primarily due to multiple inversions of genome segments at insertion sequences [12]. When comparing CA88 and CO92, IS elements identified in CA88 inside and flanking the inversions included IS*100*, IS*1541A*, IS*200G*, IS*1661*, and IS*285*. All nucleotide BLAST hits possessed greater than 99.6% identity over the full IS element lengths. Further information regarding the IS elements flanking each large rearrangement and the number of IS elements found internal to these LCBs is provided in Table 2.

**Table 2:** IS element analysis of the seven large regions of difference (RDs).

RD	Left Flank (Family)	Right Flank (Family)	Internal IS Elements in CA88
1	IS*100* (IS*21*)	IS*100* (IS*21*)	1
2	IS*100* (IS*21*)	IS*100* (IS*21*)	6
3	IS*285* (IS*256*)	IS*285*/IS*100* (IS*256*/IS*21*)	14
4	IS*100* (IS*21*)	IS*1541A*/*200G* (IS*605*)	10
5	IS*1661* (IS*3*)	IS*100* (IS*21*)	2
6	IS*100* (IS*21*)	IS*1661* (IS*3*)	7
7	IS*1661* (IS*21*)	N/A	1

We attempted to determine the ancestral states for each of the six inversions by comparing CO92 and CA88 to all sequenced *Y. pestis* genomes in GenBank. Unfortunately the large LCBs were severely rearranged in all other GenBank whole genome sequences. This is not unexpected, as *Y. pestis* 1.ORI and 2.MED last shared a common ancestor ∼7,000 years ago [4]. Even a more closely-related genome, *Y. pestis* Antiqua, shows significant rearrangement among the inverted CO92-CA88 LCBs [13]. To determine the ancestral state of these inversions, sequence from another member of *Y. pestis* 1.ORI is needed. Although sequences from three strains meeting this requirement are available as unfinished sequences in GenBank, they are not yet complete enough to obtain an accurate assembly solely from the available contigs. We expect to be able to identify possible homoplasy, determine ancestral state, and gain further resolution within the 1.ORI group and North American *Y. pestis* if these sequences ever get to a completed stage.

The magnitude of rearrangements observed between CO92/CA88 and other completed *Y. pestis* strains is not surprising given the number of IS elements detected inside each LCB. These IS elements provide a powerful mechanism for subsections of each LCB to translocate and/or invert independent of the entire block. Inversions are recognized as one of the most frequent rearrangement types in γ-proteobacteria [14]. The LCB profile of CA88 can be transformed to match that of CO92 in only ten inversion steps and zero translocations. Although there are many ways CA88 and CO92 could have evolved after diverging from a common ancestor, the ten inversion steps shown in Table 3 represent the most parsimonious solution. These results are in line with expectations, as more inversion events than translocation events are expected in recently-diverged strains of γ-proteobacteria such as *Y. pestis* [14].

**Table 3:** Output from GRIMM showing the most parsimonious reversal scenario.

Step	Description	LCB Order
**0**	**CO92**	1 2 3 4 5 6 7 8 9 10 11 12 13 14 15
**1**	**Reversal**	1 **-2** 3 4 5 6 7 8 9 10 11 12 13 14 15
**2**	**Reversal**	1 -2 3 **-4** 5 6 7 8 9 10 11 12 13 14 15
**3**	**Reversal**	1 -2 3 -4 5 **-6** 7 8 9 10 11 12 13 14 15
**4**	**Reversal**	1 -2 3 -4 5 -6 7 8 9 10 11 **-12** 13 14 15
**5**	**Reversal**	1 -2 3 -4 5 -6 7 **-9 -8** 10 11 -12 13 14 15
**6**	**Reversal**	1 -2 3 -4 5 -6 7 -9 -8 10 **-14 -13 12 -11** 15
**7**	**Reversal**	1 -2 3 -4 5 -6 7 -9 -8 **14 -10** -13 12 -11 15
**8**	**Reversal**	1 -2 3 -4 5 -6 7 -9 -8 **13 10 -14** 12 -11 15
**9**	**Reversal**	1 -2 **9 -7 6 -5 4 -3** -8 13 10 -14 12 -11 15
**10**	**Reversal (CA88)**	1 -2 **8 3 -4 5 -6 7 -9** 13 10 -14 12 -11 15

Each number represents an LCB calculated by MAUVE between CA88 and CO92. Changes between steps are underlined. Negative numbers represent an inverted LCB.

Plague was introduced into North America within the past 125 years, so any rearrangements between CO92 and CA88 have occurred within a very short time period. Ten inversions separate *Y. pestis* CO92 from CA88 but only fourteen inversions separate *Y. pestis* CO92 from KIM [14]. This suggests that the fixation rate of inversion events could be particularly high in North American *Y. pestis* strains, although any confirmation would require additional whole-genome sequences from North American strains. Fixation of inversion events in bacterial genomes during evolution is an irregular phenomenon with periods of stasis and others of acceleration [14], so our findings indicate a possible acceleration of inversion fixation rate in a species prone to inversion events and already believed to be undergoing rapid evolution.

### IS element comparison

A comparison of total IS elements in CA88 and CO92 is shown in Table 4. These totals appear to be essentially unchanged between the two genomes with many of the same interruptions and truncations observed by Parkhill et al in CO92 [9]. *In silico* analysis indicates CA88 has an additional complete copy of IS*285* as well as an additional partial copy that is missing the first 338 bases and located 69 bases downstream of an intact IS*285* element. An IS*285* segment containing only the first 338 bases was not found using BLAST.

**Table 4:** IS element counts in CO92 and CA88.

IS Element Type	CO92	CA88
IS*100*	44	44
IS*1541*	66^1^	66
IS*1661*	9	9
IS*285*	21	22^2^

1 Parkhill et al (2001) found 66 copies of IS*1541*. We only found 65 in this genome using blastn.

2 CA88 also contains a partial IS*285* copy missing the first 338 bases.

We also *in silico*-typed CA88 based on IS elements using the system described in Achtman et al [5]. Amplicon sequences were extracted and the presence/absence of IS*100* was determined for each loci. The CA88 profile matched that of CO92 using these methods and the results are shown in Table 5.

**Table 5:** Presence/absence of IS*100* loci described in Achtman et al (2004).

Strain	Y14	Y23	Y30	Y32	Y33	Y36	Y37	Y40	Y42	Y44	Y45
CA88	X	X	X	X	X	-	-	-	-	-	-
CO92^1^	X	X	X	X	X	-	-	-	-	-	-

1 CO92 profile from Achtman et al (2004).

### SNPs

15 non-synonymous and 5 synonymous SNPs were discovered when comparing the CO92 and CA88 genomes. Details for each SNP are presented in Table 6. Five of the SNPs were previously discovered between CO92 and *Y. pestis* FV-1 and these SNPs have been verified in a laboratory setting. All other SNPs have been identified *in silico* but have not been confirmed via wet bench techniques. Of the 20 discovered SNPs, the CA88 and FV-1 states matched in 15 cases.

**Table 6:** Putative non-synonymous and synonymous SNPs found between CO92 and CA88.

CO92 Position	Type	CA88 State Shared with FV-1?	Gene	CO92/CA88 (bases)	CO92/CA88 (AA)	ID from Touchman et al (2007)
4,225	non-synonymous	NO	YPO0005	A/T	V/E	-
351,821	non-synonymous	YES	YPO0342	T/G	H/Q	-
471,201	non-synonymous	NO	YPO0449	C/A	C/F	-
917,155	non-synonymous	YES	YPO0837	A/G	S/G	-
1,939,841	non-synonymous	YES	YPO1701	A/G	L/P	-
2,273,616	non-synonymous	YES	YPO2000	G/C	T/R	-
2,278,317	non-synonymous	YES	YPO2005	A/G	V/A	m
2,300,659	non-synonymous	YES	YPO2029	T/G	D/A	p
2,619,611	non-synonymous	YES	YPO2328	T/G	E/A	q
3,608,932	non-synonymous	YES	YPO3243	T/C	D/G	-
3,647,867	non-synonymous	YES	YPO3273	C/T	A/V	-
3,655,609	non-synonymous	YES	YPO3275	T/C	K/E	-
3,789,780	non-synonymous	NO	YPO3393	A/G	W/R	-
4,579,183	non-synonymous	YES	YPO4060	A/G	S/G	s
4,624,135	non-synonymous	YES	YPO4103	C/G	P/R	-
150,946	synonymous	YES	YPO0138	C/A	-	-
1,939,828	synonymous	YES	YPO1701	T/G	-	-
3,394,022	synonymous	NO	YPO3352	C/T	-	-
3,739,401	synonymous	YES	YPO3481	C/A	-	r
3,886,839	synonymous	YES	YPO3040	T/C	-	-

None of the non-synonymous SNPs result in nonsense mutations, indicating that no pseudogenes were created via this method. Gene gain/loss does not appear to be occurring between CO92 and CA88, but only approximately 125 years have passed since CO92 and CA88 shared a common ancestor. As these two strains were both found in Western North America, one can hypothesize that essential genes in CO92 are also essential in CA88 and that evolutionary pressure to diverge due to environmental conditions would be minimal in such a short time period.

### Whole gene differences

No obvious whole-gene differences were found when comparing CO92 and CA88. Several small regions of non-shared sequence were detected by MAUVE, but in many cases these regions matched transposases. Because the BLAST method showed only a difference of one IS element between CO92 and CA88, MAUVE likely found IS elements that have shifted positions and was unable to match them to their counterparts in the other strain.

### Plasmid comparisons

In addition to the chromosome, we also examined differences between the plasmids of CO92 and CA88. LCB profiles matched exactly for both pMT1 and pCD1 but a 999-bp alignment gap was discovered in pPCP1 of CA88. This gap corresponds to nucleotide positions 3124-4122 in the CO92 pPCP1 plasmid sequence (GenBank accession: AL109969). No annotated features appear in the corresponding CO92 pPCP1 region but this region is flanked by the *rop* and *pim* genes (YPPCP1.03 and YPPCP1.04). Both *rop* and *pim* are intact in the CA88 pPCP1 sequence and the location of the gap corresponds to the ColE1 site in the CO92 plasmid sequence. ColE1 is the origin of replication for the pPCP1 plasmid in *Y. pestis*. This alignment gap was also present when comparing CA88 to Nepal516 (2.ANT, [13]), Antiqua (1.ANT, [13]), KIM (2.MED, [12]), 91001 (0.PE4, [15]) and CO92 (1.ORI, [9]). Confirmation via PCR shows that the alignment gap in CA88 is not real. This region may have been missed during sequencing and was not included as part of the draft CA88 pPCP1 contig sequence.

### Summary

Initial findings suggest that North American *Y. pestis* strains may be undergoing inversion fixation at relatively high rates considering the short time span separating the CA88 and the CO92 isolates. Differences in IS element copy number were observed, as well as several SNPs between CO92 and CA88. No whole-gene differences were detected using the CA88 contig sequences We hope these initial findings will encourage the scientific community to fully-sequence more *Y. pestis* strains from North America and abroad, as it would further our understanding of the evolutionary history of this important pathogen.

## Materials and Methods

### DNA Preparation and Sequencing CA88

DNA preparation was performed via chloroform extractions [16], [17]. The genome was sequenced at the Joint Genome Institute (JGI) using small (2–3kb) and medium (6–8kb) insert plasmid libraries. Draft assemblies were based on 15× coverage. The Phred/Phrap/Consed software package (http://www.phrap.com) was used for sequence assembly and quality assessment [18]. After shotgun sequencing, reads were assembled with parallel phrap (High Performance Software, LLC). Two rounds of finishing were performed resulting in 10 contigs and 6 scaffolds. During finishing, possible mis-assemblies were corrected by transposon bombing (Epicentre Biotechnologies) of bridging clones. Gaps between contigs were closed by editing in Consed, by custom primer walks, or by PCR amplification.

### Assembling the contigs into a pseudomolecule

Ten contigs were obtained, of which three contigs contained plasmid sequences. The seven contigs comprising the *Y. pestis* CA88 chromosome were aligned to the complete genome sequence of *Y. pestis* CO92 (GenBank accession: NC_003143) using MAUVE [19]. After determining proper contig order using MAUVE, the seven contigs were concatenated into one pseudomolecule representing the CA88 chromosome.

### Identifying flanking transposases

Once the CA88 pseudomolecule was produced, it was aligned against the whole-genome sequence of CO92 and the positions of the major LCBs were determined relative to CO92. The CO92 GenBank annotation was queried to identify annotated coding sequences (CDS) immediately flanking LCB boundaries.

### Locating additional IS elements inside rearranged LCBs and throughout the genome

Nucleotide sequences for IS elements were obtained from IS Finder (http://www-is.biotoul.fr/) by searching for “pestis” in all fields. This returned a list of IS elements documented in *Y. pestis* and *Y. pseudotuberculosis*. Sequences for each IS element were saved and compared to a sequence database containing CA88 nucleotide sequences for the six inversions and the largest rearrangement. The comparison was performed using BLAST [20].

The same procedure was used to locate IS elements on a whole-genome scale. IS element sequences were queried against the CO92 genome and the CA88 pseudomolecule using blastn and the results were compared.

### SNP discovery

A high-throughput automated bioinformatic pipeline was used to discover and classify SNPs. This pipeline integrates the whole-genome alignment tools MUMmer to map contigs of the draft genome to the reference genome sequence and to identify putative polymorphic sites [21]. Base-calling software assigns an error probability to each base pair in a sequenced read. The probability of each underlying sequence is used to compute the accuracy of any base pair in the assembled genome. High quality SNPs were selected based on a combination of these statistics and coverage information. To limit the number of false positives due to sequencing errors, the comparative analysis in this study used only regions with at least 3× coverage where the chromatograms agreed with each other and the median quality was more than 30. For each high quality SNP that was located within a gene, the effect of the nucleotide change on the encoded protein was reported, which allowed us to differentiate between synonymous and non-synonymous SNPs. All duplicated regions were removed from consideration. In this comparative analysis, it was assumed that each base pair of CO92 was of high quality, as the underlying chromatograms for the consensus sequence was not available and this genome is closed.

### Whole gene differences

The islands file created by MAUVE was used to determine regions potentially unique to each genome. For regions identified as being solely present in CA88, sequence was extracted from the appropriate positions within the pseudomolecule and queried against NCBI's nr database using the blastx algorithm. For regions identified as being solely present in CO92, positions from the MAUVE islands file were compared to the CO92 GenBank annotation.

### Determining the most parsimonious rearrangement scheme

The GRIMM website (http://nbcr.sdsc.edu/GRIMM/mgr.cgi) was used to analyze the LCB patterns produced in MAUVE. A signed analysis using circular chromosomes was selected. GRIMM analyzes LCB-order and orientation to determine the most parsimonious method to transform one strain into another [22].

### Plasmid comparisons

All plasmids were compared to their CO92 counterparts using the MAUVE Aligner. In the event of gaps between detected LCBs, sequence for this region was extracted from the appropriate strain and compared to its counterpart via BLAST to confirm the findings from MAUVE. PCR was run on CA88 and CO92 to confirm the existence/absence of a sequence gap in the pPCP1 plasmid of CA88. Primers were designed on each side of the putative gap. The following primer sequences were used: CA88-for 5′-AAG CCA GAG CCT GAT ACT GCT TGA-3′ and CA88-rev 5′AAG TAA CAT GGG TGT TAC CGC AGC-3′.
